# Evaluation of allelopathic potentialities of an invasive taxon, *Mesosphaerum suaveolens* (L.) Kuntze, and bio-assay-guided identification of the involved allelochemicals

**DOI:** 10.1038/s41598-026-43350-w

**Published:** 2026-03-26

**Authors:** Agamani Pattanayak, Parthapratim Maiti

**Affiliations:** Plant Taxonomy and Ecophysiology Laboratory, Department of Botany (U.G. and P.G.), Midnapore College Autonomous, Midnapore, West Bengal India

**Keywords:** Allelochemicals, Alternative herbicide, Crop science, In-silico analysis, Mesosphaerum suaveolens, Sustainable agri-management, Biochemistry, Biotechnology, Plant sciences

## Abstract

Weeds in crop fields pose a serious threat and devastate the overall agro-economy. Popular weed management techniques include manual and mechanical tillage, as well as the application of synthetic herbicides. These are used worldwide but have tremendous harmful side effects on both human health and environmental sustainability. So, alternative techniques are in great demand. The use of invasive alien species, with allelopathic potentialities against obnoxious weeds, has emerged as an alternative strategy for the formulation of environmentally safe bioherbicides. In the present investigation, extracts of an invasive species, *Mesosphaerum suaveolens* (L.) Kuntze was evaluated for their allelopathic effects on three test species *Vigna radiata* (L.) Wilczek., *Sorghum bicolor* (L.) Moench, and *Parthenium hysterophorus* L. Hexane-ethyl acetate fraction (1:1 and 1:2) of the methanolic leaf extract of *M*. *suaveolens* exhibits maximum inhibition. Allelochemicals affected the T_50_ (time required for 50% germination of the total seeds) value of the bioassay materials in reference to the control. Biochemical analysis of the seeds treated with allelochemicals elucidated the reduction in insoluble carbohydrates, nucleic acids, protein-amino acid contents, and amylase action. The standard physical parameters also get affected. An increase in the stress-related antioxidant defence enzymes- catalase (166.81%), superoxide dismutase (242.02%), peroxidase (80.19%), as well as soluble carbohydrate (131.53%) was observed in both the treated seeds and seedlings. The pigment profiles, i.e., chlorophyll a, b, and carotenoids, changed. Proline contents get altered (102.43%). There was a significant change in the levels of indole acetic acid (47.77%), gibberellin (52.23%), and nitrites (182.62%) in the allelochemical-treated seedlings. Bioassay-guided fractionation with GC-MS analysis identified the major allelochemicals as 3, 4, 5-trihydroxybenzoic acid, transferulic acid, chlorogenic acid, sabinene monohydrate, and p-coumaric acid. In-silico analysis showed that the reported allelochemicals exhibit strong binding affinities. Chlorogenic acid (− 6.24 and − 6.32 kcal/mol) interacted prominently with tryptophan synthase β-subunit (PDB: 5DW3) and auxin-binding protein 1 (PDB: 1LRH), while caryophyllene oxide (-6.91 kcal/mol) modulated the activity of 4-hydroxyphenylpyruvate dioxygenase (PDB: 6J63). The present findings firmly establish the allelopathic potential of the invasive herb *M.* suaveolens as a robust, eco-friendly bioherbicide and provide clear direction for the sustainable management of the alien species.

**Supplementary Information:**

The online version contains supplementary material available at 10.1038/s41598-026-43350-w.

## Introduction

 Weeds pose a serious challenge to agriculture, that compete directly with crops for vital resources and gradually exhausting nutrients from fertile soils. This competition ultimately reduces both the quality and quantity of harvested product that affects the overall agro-economy^1^. When weeds and food crops grow simultaneously, they contend for light, water, and space, that often give a competitive advantage to weeds^2^. As a result, weed control has become an essential component of modern agriculture. Now a days chemical herbicides have become the most widely adopted method for managing weeds across the world, largely because they are cost-effective, time-efficient, and require less labour compared to manual or mechanical practices^3–6^.

Continuous and long-term use of synthetic herbicides on the same land has created new problems, which include herbicide resistance, chemical residues in crops, and disruption of soil biochemical processes. Continuous exposure can diminish populations of beneficial soil microorganisms and contribute significantly to environmental degradation. Herbicide residues may enter the food chain, where they bioaccumulate and interfere with human cellular processes^7,8^.

Such effects have become a major public health concern, as they can trigger various cellular irregularities. For example, glyphosate, a commonly used herbicide, is associated with the development of malignant tumours, including cholangiocarcinoma and non-Hodgkin lymphoma. These challenges highlight the urgent need for safer, sustainable alternatives to conventional herbicides.

Plant-derived phytochemicals with natural herbicidal or phytotoxic properties have garnered increasing interest, as they are generally biodegradable and they do not leave harmful residues^9,10^. Many invasive alien plant species possess strong allelopathic abilities which make them proper promising candidates for bioherbicide development^11–15^. Allelopathy is the chemical interactions between plants which is mediated through leaching, root exudation, or decomposition of plant materials^16,17^. The responsible compounds are known as allelochemicals. They influence the growth, metabolism, or behaviour of neighbouring plants, either positively or negatively^18–20^. Although allelopathic interactions occur widely across plant groups, many of the strongest effects have been reported in invasive species^21–25^.

Exotic plants often outcompete native vegetation and establish dense, monospecific strands by releasing allelochemicals^26–28^. These plants typically synthesise a rich spectrum of allelochemicals-phenolics, terpenoids, alkaloids etc., which can be exploited in weed-management strategies^20,29^. Research on the allelopathic potential of plants is expanding rapidly, especially with the aim of developing eco-friendly approaches to weed control^24,30^. Allelochemicals may be used alone or combined with reduced herbicide doses, intercropping, crop rotation, soil application, cover cropping, or green manuring^31,32^. These compounds can interfere with germination, inhibit root or seedling growth, reduce photosynthetic efficiency, hinder enzyme activities, disrupt nutrient uptake, and impair crucial cellular processes such as respiration, protein synthesis, and cell division^33,34^.

*Mesosphaerum suaveolens* (bush mint or Vilyati tulsi) is tropical and subtropical weed with known medicinal properties and known allelopathic activities. Previous studies have reported its inhibitory effects on various species like pea, wheat, cress, lettuce, alfalfa, rapeseed, timothy, crabgrass, barnyard grass, Italian ryegrass, and ragi^13,35–37^. The influence of *Mesosphaerum* on mung bean under Indian climatic conditions has not been explored yet. Phytochemical analyses of *M*. *suaveolens* have revealed essential oils rich in phenols, flavonoids, terpenes, and alcohols^38,39^.

The test crop *Vigna radiata* (mung bean/green gram), is an economically valuable member of the Fabaceae family, widely cultivated across tropical and subtropical regions of Asia, Africa, South America, and India. This annual plant typically reaches 30–100 cm in height and completed its life cycle within 60–70 days. Its compound racemose inflorescence and pod-like fruits are characteristic features, and the crop is recognized for its high protein content and nutritional importance^40^. *V*. *radiata* plays a key role in crop rotations and soil fertility^41^. It also holds significance in traditional medicine due to its antimicrobial, antioxidant, anti-inflammatory, and antidiabetic attributes^42^.

*V. radiata* is widely used as a model organism in allelopathy research because of its rapid growth, short life cycle, high sensitivity to phytotoxins, uniform germination, and reproducible results. These traits make it ideal for assessing both inhibitory and stimulatory effects of allelochemicals under laboratory and greenhouse conditions^43–46^. For these reasons, it was selected to assess the allelopathic effects of *M*. *suaveolens* in the present study.

This work aims to: (a) evaluate the allelopathic potential of the invasive weed *Mesosphaerum suaveolens* against the crop plants *Vigna radiata*, *Sorghum bicolor*, and the weed *Parthenium hysterophorus*: (b) assess the physical and biochemical responses of treated seeds and seedlings; (c) conduct bioassay-guided fractionation to identify the active allelochemicals; and (d) perform in-silico analyses of these compounds against three target species-Auxin Binding Protein 1 (1LRH), 4-hydroxyphenylpyruvate dioxygenase (6J63), and Tryptophan Synthase β-subunit (5DW3)- to propose potential; modes of action.

## Materials and methods

### Plant materials

Disease-free leaf samples of *M*. *suaveolens* (L.) Kuntze. were collected from adjacent areas of the Kangsabati river bank, Midnapore, Paschim Medinipur, West Bengal, India (22.4257 °N, 87.3199 °E). Leaves were washed thoroughly under running tap water. Seeds of bioassay materials of *Vigna radiata* (L.) Wilczek, *Sorghum bicolor* (L.) Moench, and *Parthenium hysterophorus* L. were purchased from the local market and collected from the surrounding areas, respectively.

### Extraction of allelochemicals

Six solvents (methanol, ethanol, chloroform, diethyl ether, n-hexane, ethyl acetate; HiMedia, India) were used. The methanol extract exhibited the highest allelopathic potential. A total of 250 g of shade-dried powdered leaves was mixed with 500 mL of methanol (500 mg mL^−1^) and stirred for 48 h. The mixture was filtered through Whatman No. 1 paper. The filtrate was concentrated using a rotary evaporator under low pressure and lyophilised. 5 g of lyophilised extract were fractionated using silica gel column chromatography (200 g silica, 100 × 3.5 column), eluting with hexane-ethyl acetate mixtures in different ratios (1:9, 2:8, 3:7, 4:6, 1:1, 3:1, 7:3, 8:2, 9:1, 1:2, 1:3). Fractions (10 mL each) were collected. Other solvents were tested, but did not provide distinct separations. Solvents were evaporated, and dried residues were dissolved in 10 mL of double-distilled water for bioassays.

### Bioassay-guided partial purification of the allelochemicals

All 11 fractions were assessed using *V*. *radiata* seeds following ISTA^47^. Surface-sterilised seeds were placed in 90-mm Petri dishes (Borosil). For every treatment, 10 seeds per Petri plate were taken. Three sets were prepared for each experiment. The experiment was repeated three times. Seeds were pre-soaked overnight in each fraction (10 mL, 0.5 mg mL^−1^) and incubated at room temperature. Controls contained double-distilled water only.

**Determination of T**_**50**_.

T_50_ values were calculated using standard methodology^48^. Germination percentage was recorded every 24 h up to 120 h^49^. Percentage inhibition was calculated as; Inhibition (%)= [1 − (sample extract/control)] x 100.

**TTC Viability Assay**.

Dehusked seeds (100 seeds per treatment) were incubated in 0.5% 2, 3, 5-triphenyl tetrazolium chloride (TTC) in the dark. The percentage of TTC-stained seeds (red) was recorded^50^.

**GC-MS analysis**.

Hexane-Ethyl acetate (1:1) fraction exhibited the highest inhibitory effect, followed by H: EA (1:2), and was further used to evaluate different physiological parameters of the treated bioassay materials. The major fragments were subjected to Gas Chromatography and Mass Spectrometry (GC-MS-QP2010 Ultra, Agilent) analysis. The instrument was configured with a DB-5 Ultra Inert column (30 m in length and 0.25 mm inner diameter) and a flow rate of 1.21 mL/min. A 2 µL sample was injected in split mode at an injection temperature of 260 °C. The ion source temperature was kept at 220 °C. m/z ratio scanning was performed from 40 to 650 for 35 min^51^. Ionisation current and ionisation energy were kept at 0.1 kV and 70 eV, respectively, and the ionisation source was maintained at 250 °C. The mass fragmentation pattern was analysed using X-Calibur software. The injector port and oven temperature were maintained at 240 °C and 50 °C, respectively. The ramping time was 10 °C min^−1^ up to 260 °C, and helium was used as the carrier gas. The compounds were identified based on the similarity index (SI) and reverse similarity index (RSI) values obtained from the National Institute of Standards and Technology (NIST) library, and their chemical formula and probability percentage were presented.

### Effect of allelochemicals on the physical and biochemical parameters of the bioassay materials

#### Sample preparation

Mung bean (*Vigna radiata*) seeds were treated with allelochemicals for 24 h. Ten seeds were used per biochemical assay, with three biological replicates and three technical replications per assay. Seedlings for physiological assays were grown in plastic pots until 15 days old and then exposed to allelochemical fractions for 2 weeks (foliar + drenching; 10 mL twice daily), followed by a 7-day recovery period. Hexane-ethyl acetate fractions (1:1, 1:2, 1:3) were dried, weighed, and re-dissolved in double-distilled water to 0.5 mg mL^−1^ for application.

#### Biochemical Assays in seeds

**Free amino acids**.

Free amino acids in seed leachates were estimated by the ninhydrin method^52^. Seeds were soaked in 10 mL of distilled water for 24 h. 1 mL of leachate was mixed with 3 mL of 0.1% ninhydrin (in 80% ethanol) and incubated at 100 °C for 15 min. After cooling, the volume was adjusted to 4 mL with 80% ethanol, and absorbance was recorded at 580 nm. Quantification used glycine standards.

**Soluble carbohydrates**.

Soluble carbohydrates were determined using the anthrone method^53^. 1 mL of diluted leachate was mixed with 3 mL of precooled 0.2% anthrone reagent (in concentrated H_2_SO_4_) and incubated for 30 min. Absorbance was taken at 610 nm and quantified using glucose standards.

**Insoluble carbohydrates**.

The ethanol-insoluble residue from seed extracts was hydrolysed with 5 mL 25% H_2_SO_4_ at 80 °C for 30 min. 1 mL of the hydrolysate (appropriately diluted) was reacted with anthrone reagent as above and measured at 610 nm.

**Protein**.

Proteins from 100 mg seed kernels were extracted in 80% ethanol, washed sequentially with 10% TCA, ethanol, ethanol: chloroform (3:1), and ether, dried, and solubilised in 0.5 N NaOH at 80° C for 1 h. Protein content was estimated at 650 nm using the Lowry method^54^ with BSA standards.

**Nucleic acids**.

**DNA**.

DNA was extracted from 100 mg of seed kernels using 5% perchloric acid. 1 mL of extract was reacted with 5 mL of diphenylamine reagent (diphenylamine in glacial acetic acid and concentrated H_2_SO_4_) in a boiling water bath for 30 min. After cooling, absorbance was recorded at 610 nm. DNA content was determined from a herring sperm DNA standard curve^55^.

**RNA**.

For RNA, 3 mL of perchloric acid extract was mixed with 3 mL freshly prepared orcinol reagent (1 g orcinol in concentrated HCl with 0.1% FeCl_3_.6H_2_O), boiled for 20 min, and measured at 700 nm^56^. Quantification used yeast RNA standards.

**Amylase activity**.

Amylase activity was estimated following^57^. Crude enzyme (supernatant for seed kernels homogenised in 0.1 M phosphate buffer, pH 6.5) was incubated with 0.1% soluble starch in sodium acetate buffer (pH 5.0) at 37 °C for 10 min. Reaction was stopped with iodine-HCl solution, and the absorbance of the starch-iodine complex was read at 620 nm.

**Peroxidase activity**.

Peroxidase activity was measured using guaiacol as substrate^58^. Seed kernels were homogenised in 0.05 M sodium phosphate buffer (pH 6.5), centrifuged at 10,000 g and the supernatant was used. Reaction mixture contained buffer, 2% H_2_O_2_, 2% guaiacol, and 0.1 mL enzyme extract. Formation of tetraguaiacol was monitored at 470 nm.

**Catalase activity**.

Catalase activity was measured by monitoring the decomposition of H_2_O_2_ at 240 nm^59^. Seed kernels were homogenised in 0.1 M phosphate buffer (pH 6.5), centrifuged twice, and the supernatant was used. Reaction mixture contained phosphate buffer, 2% H_2_O_2_, and enzyme extract. Activity was expressed as units mg^−1^ fresh weight.

#### Physical and physiological parameters in seedlings

##### Morphometric measurements

Seedlings treated with allelochemicals were analysed for stem and root length, leaf number, leaf area, fresh and dry biomass, and pod length and breadth.

##### Biochemical assays in seedlings

**Photosynthetic Pigments**.

Chlorophyll a, chlorophyll b, and carotenoids were quantified from 1 g of leaves extracted in 80% acetone^60^. Absorbances were measured at 663, 645, and 480 nm, and pigment levels calculated using:

$${\text{Chl a}}\, = \,12.27A_{{663}} \, - \,2.69A_{{645}}$$

$${\text{Chl b}} = {\text{ 22}}.{\mathrm{9A}}_{{{\mathrm{645}}}} - {\text{ 4}}.{\mathrm{68A}}_{{{\mathrm{663}}}}$$

$$Carotenoids = A480- (0.114A663-0.638A645)$$

**Proline**.

Proline levels were measured using the acidic ninhydrin method^61^. Leaves (100 mg) were homogenised in 3% sulfosalicylic acid, reacted with acidic ninhydrin and glacial acetic acid, and extracted into toluene. Absorbance of the organic phase was read at 520 nm.

**Lipid Peroxidation (MDA)**.

MDA content was estimated using the TBA reaction^62^. Leaf extracts in 0.1% TCA were mixed with 10% TCA and 0.25% TBA, incubated at 95 °C for 30 min, cooled, and absorbance was taken at 532 and 600 nm. MDA concentration was calculated using ε = 155 mM^−1^ cm^−1^.

**Superoxide Dismutase (SOD)**.

SOD activity was measured via NBT photoreduction assay^63^. Leaf extracts in phosphate buffer (pH 7.8) + EDTA were mixed with methionine, NBT, riboflavin, and EDTA, and incubated under light. Absorbance was measured at 560 nm. 1 unit was defined as 50% inhibition of NBT reduction.

**Peroxidase and Catalase**.

Peroxidase and catalase were measured from seedling leaves using the same protocols described for seeds^58,59^.

**Phytohormones and Nitrite**.

**Indole-3-Acetic Acid (IAA)**.

IAA was extracted from 200 mg of leaf tissues in 80% methanol and reacted with Salkowski reagent^64^. Absorbance of the resulting pink chromophore was recorded at 520 nm, and concentration expressed as ng g^−1^ FW.

**Gibberellic Acid (GA**_**3**_**)**.

GA3 was determined using the 2,4-DNP method^65^. Methanolic leaf extract was reacted with 2,4-DNP, heated to 100 °C, cooled rapidly, alkalised with KOH-methanol, diluted with water, and the absorbance was recorded at 430 nm. Quantification used GA_3_ standards.

**Nitrite**.

Nitrite levels were estimated using the sulfanilamide-NED coupling reaction^66^. Leaf extracts were clarified with zinc acetate, centrifuged, and reacted with sulfanilamide followed by NED. Absorbance was measured at 546 nm and compared with KNO_3_ standards.

### Molecular docking studies to assess the binding interaction between the allelochemicals and the auxin-binding protein 1-ABP1 (PDB ID: 1LRH), 4-hydroxyphenylpyruvate dioxygenase (6J63), and Tryptophan Synthase beta-subunit (5DW3)

The potential mechanism of phytotoxic action of allelochemicals was assessed through molecular docking studies. It was conducted between identified allelochemicals and the above-mentioned target receptors. The structure of the target receptor protein was retrieved from the Protein Data Bank database (PDB ID). The 3D Structure of allelochemicals was acquired from the PubChem database. Molecular docking studies were done with AutoDock Vina. Initially, water molecules, ions, and previously bound ligands were removed from the structure of the protein. Polar hydrogen and Kollman charges were added using AutoDockTools-1.5.7. Grid preparation was done by covering the entire receptor molecule. To obtain the best docking confirmation, the ligand was put in a flexible form. PyMol was used for the docking analysis.

### Statistical analysis

All experiments were performed in triplicate, and the results are presented as means ± standard errors (SE). Data were analysed by Prism GraphPad software version 9.2.0 (332) (San Diego, CA, United States). Tukey’s multiple comparison test was performed to evaluate the valid statistical difference between the datasets.

## Results and discussions

### Allelochemicals alter the growth and germination of Mung plants

**Fig. 1:**
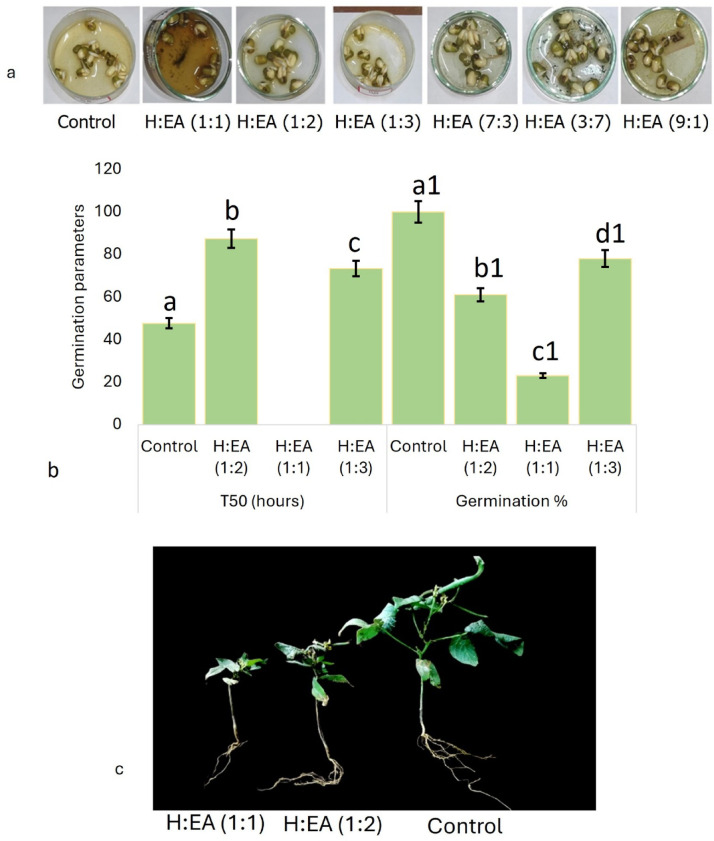
Effect of different fractions of allelochemicals on the seed germination profiles of **a**-mung bean, **b**- germination parameters (T_50_ values, and TTC staining assay), and on the **c**- growth of mung bean seedlings. The values in the graph (**b**) are the mean ±standard error of three independent replicates. The different letters mentioned on the graphs represent the valid statistical differences among them, i.e., **a**, **b**, and **c** indicate the difference in T_50_ values among the control and treated sets, whereas **a1**, **b1**, **c1**, and **d1** for germination percentage indicate valid differences among them. Tukey’s multiple comparison test was performed to analyse the results (*p* < 0.05).

The allelochemicals inhibited the germination of mung bean seeds, and the treated set exhibited only 32% of germination, whereas the control set exhibited 98.71% of germination (Fig. 1a–c). The stunted growth of the plant parts, reduced dry weight, yellowing and wilting of leaves, drooping of leaf tips, the appearance of necrotic lesions, and premature senescence were due to inhibition of cell division, degradation of pigments, infrequent stomatal opening, and inadequate supply of nutrients triggered by the allelochemical application^67–69^. The effect of allelochemicals on the physical conditions of the mung bean seedlings is recorded in Table 1 (Fig. 1d). Allelochemicals, after being released into the soil, cause toxicity in the surroundings, which leads to metabolic poisoning of the treated plants and causes shortening of root and shoot length^70^. Also, ion transport, i.e., mineral nutrition and plant water relationship, is hindered, which ultimately reduces the fresh biomass^71^. The investigational outcomes of^67,72^, dealing with *Mangifera indica*, *Cyperus rotundus*, and sesame, respectively, match our results. Allelochemicals block the series of enzymatic intermediates (α-amylase, phosphorylase, etc.) necessary for seed germination^73^. They choke the normal rates of seed germination, decrease vigour, and reduce radicle length and dry weight. They essentially restrict the vertical growth and cell division of the root system, which affects plants’ nutrient absorption profile. The investigational results of^74–78^ in the case of *Echinochloa crusgalli*, *Leucaena leucocephala*, *Miscanthus sacchariflorus*, *Sesamum indicum*, *Prosopis juliflora* coincide with our data.

**Table 1:** Effect of *M*. *suaveolens* allelochemical on the standard physical parameters of the mung bean seedlings.

	Control		1:2		1:1		1:3	
	30 days	60 days	30 days	60 days	30 days	60 days	30 days	60 days
Root length (cm)	4.52 ± 0.61	13.59 ± 0.97	1.55 ± 0.11	3.75 ± 0.23	1.39 ± 0.07	8.17 ± 0.74	1.92 ± 0.18	10.2 ± 0.80
Shoot length (cm)	14.66 ± 0.93	32.55 ± 1.47	8.67 ± 0.29	21.20 ± 0.91	7.29 ± 0.18	20.14 ± 0.93	8.16 ± 0.35	22.09 ± 1.12
Leaf no	3.33 ± 0.57	6.67 ± 1.15	1 ± 0	4.33 ± 0.57	1 ± 0	3.33 ± 0.57	1 ± 0	5.33 ± 0.57
Leaf area (mm^2^)	1158 ± 56	1673 ± 59	415 ± 32	612 ± 39	320 ± 24	512 ± 31	470 ± 31	683 ± 58
Stem circumference (cm)	0.81 ± 0.08	1.92 ± 0.11	0.57 ± 0.06	1.24 ± 0.10	0.37 ± 0.03	0.99 ± 0.09	0.54 ± 0.07	1.48 ± 0.12
Fresh weight (gm)	1.44 ± 0.21	7.62 ± 1.01	0.72 ± 0.08	3.14 ± 0.39	0.55 ± 0.04	1.72 ± 0.40	0.93 ± 0.10	2.14 ± 0.33
Dry weight (gm)	0.72 ± 0.10	1.76 ± 0.23	0.18 ± 0.04	0.79 ± 0.13	0.15 ± 0.02	0.37 ± 0.09	0.26 ± 0.06	0.52 ± 0.10
No of nodule	–	28.67 ± 1.52	–	11.67 ± 0.57	–	6.67 ± 0.57	–	16.67 ± 0.57
No of flower	–	16.67 ± 0.57	–	5.57 ± 0.57	–	3.57 ± 0.57	–	8.57 ± 0.57
No of fruit	–	4 ± 0	–	1 ± 0	–	0	–	2 ± 0
Length of the pod (cm)	–	7.82 ± 0.13	–	3.43 ± 0.07	–	0	–	4.12 ± 0.09
Breadth of the pod (cm)	–	0.91 ± 0.09	–	0.32 ± 0.04	–	0	–	0.45 ± 0.06

The values are represented as the means ± Standard error (SE) of the three replicates.

Allelochemicals interfere with the cellular respiratory pathways of seedlings, i.e., the ETC of mitochondria, and ATP production is affected. The inability of the mitochondrial machinery to meet the proper energy demand is reflected in the stunted growth of seedlings^79^. An osmotic imbalance in cells causes the unavailability of water and seed germination, and basic chloroplast functions are broadly hampered^80^. Extracts of different members of Lamiaceae, i.e., basil (*Ocimum basilicum*), goldenrod (*Solidago virgaurea*), lemon balm (*Melissa officinalis*), sage (*Salvia officinalis*), and thyme (*Thymus vulgaris*), are reported to inhibit seed germination and reduce shoot and radical growth of velvet leaf weed, soybean, and maize, respectively^81–84^. *Cryptostegia grandiflora*, *Ailanthus altissima*, *Phytolacca americana*, *Reynoutria japonica*,* Alternanthera ficoidea*, and *Wedelia trilobata* block the germination and growth of *Linum usitatissimum*, *Guizotia abyssinica*, *Lactuca sativa*, *Medicago sativa*, *Bromus catharticus*, *Vigna radiata*, and *Wedelia chinensis*^15,85–88^.

Not only Mung plants, the allelopathic potentialities of the allelochemicals found in the present investigation were further estimated on two other bioassay materials, i.e., seeds of *Sorghum bicolor*, and *Parthenium hysterophorus*, and 31% (H: EA-1:1 treated), 22% (H: EA-1:2 treated) and 67% (H: EA-1:1 treated), 54% (H: EA-1:2 treated) seed germination inhibition was detected. This highlights the specificity of the allelochemicals to exhibit a higher degree of inhibition against weeds and elucidates their broad-spectrum utility. Further field-based trials, through intercropping and co-application with minimal dosages of synthetic herbicides, are required to gradually replace or reduce the dosage of synthetic allelochemicals.

### Impact of allelopathic components on the pigment profile of test plants

**Fig. 2:**
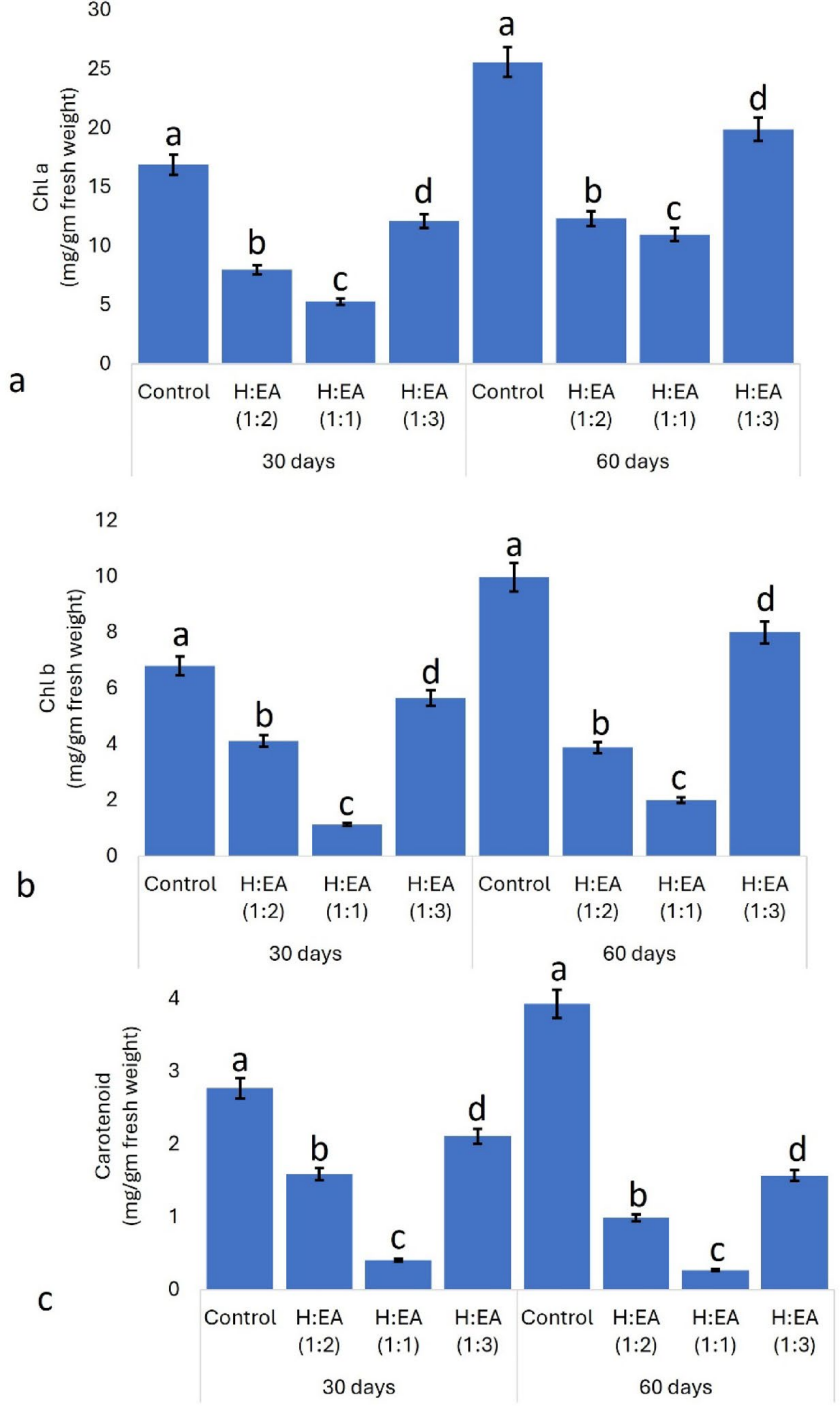
Effect of different fractions of allelochemicals on the pigment profile, i.e., **a**- Chl a, **b**- Chl b, and **c**- carotenoid content of the treated and control plants after 30 and 60 days of treatment. The values in the graph are the mean±standard error of three independent replicates. The different letters mentioned on the graphs represent the valid statistical differences among them. Tukey’s multiple comparison test was performed to analyse the results (*p* < 0.05).

Mung bean seedlings treated with H: EA (1:1 and 1:2) fraction exhibit the maximum reduction of chlorophyll a and chlorophyll b contents in both the 30-day and 60-day treatments in comparison to the control seedlings (Fig. 2a, b). There was almost a two-fold decrease in Chl-a content in both one-month (control- 16.22 ± 1.29, treated- 7.25 ± 0.98 mg/gm fresh wt.) and two months (control- 24.19 ± 1.79, treated- 10.87 ± 1.07 mg/gm fresh wt.) treatment, whereas Chl b shows a five-fold loss after thirty (control-6.87 ± 1.04, treated-1.94 ± 0.51 mg/gm fresh wt.) and sixty (control- 10.61 ± 2.07, treated- 2.91 ± 0.43) days of application of hexane-ethyl acetate (1:1) allelochemical fraction. The degradation of chlorophyll contents may be due to either the blockage of the biosynthetic pathways of chlorophyll or the triggering of chlorophyll degradation processes. Allelochemicals attack the pyrrolic ring and phytol chain of the chlorophyll molecule^89–91^. Allelochemicals primarily hinder fluorescence parameters, maximum PSII quantum efficiencies, the quantum yield of PS-II, and microcystin content, thus stunting growth^92^. A reduction in chlorophyll content hampers the photosynthetic processes, and as a result, photosynthate production (i.e., glucose and other organic acids) is hampered, and carbon skeleton, electron transport chain, and energy metabolism are disturbed simultaneously^93–95^. As a result, the plant utilises the pre-stored reserve food materials, which eventually stunt the growth of plants and hamper the normal physiological processes. Other than chlorophyll, carotenoids also perform a vital role in the reception and transformation of energy in autotrophs. They offer protection of the plant cells (i.e., photosynthetic apparatus) from photooxidative damage and act as the major component of the Light-harvesting Complex (LHC)^96^. There is a sharp change in the carotenoid contents also (control- treated after 60 days of treatment), which directly affects the electron transport system (ETS), and the plant cells face huge photooxidative stress (Fig. 2c). Oxidative degradation, damage of chloroplasts and associated degradation of thylakoid-bound carotenoids, inhibition of carotenoid biosynthesis, and downregulation of phytoene synthase and lycopene cyclase are the primary reasons for reduced carotenoid contents in the treated leaves. In short, the whole plant metabolism faces striking anomalies^97,98^. Phytotoxic metabolites are reported to inhibit the enzymatic action of 4-hydroxyphenyl pyruvate dioxygenase and/or phytoene desaturase, which are considered the key factors in carotenoid biosynthesis^99^. Allelochemicals have been randomly reported to inhibit chlorophyll biosynthetic pathways, leading to a decreased concentration of chlorophyll and stunted growth of the treated plants, e.g., *Glycine max*, and *Tignoella foenum-graceum*, respectively^100,101^. Allelochemicals produced by various organisms significantly influence the growth and physiology of other species. For instance, allelochemicals from different *Synechococcus* sp. strongly affect the growth of other blue-green algae and diatoms^102^. Similarly, compounds from *Artemisia aragyi* inhibit the growth of *Microcystis aeruginosa* by suppressing the biosynthesis of chlorophyll a (Chl a), carotenoids, and phycobiliproteins (PBPs)^103^. In higher plants, allelochemicals derived from *Brassica napus*, *Sinapis alba*, *Passiflora incarnata*, and *Mentha piperita* alter the chlorophyll a, chlorophyll b, and carotenoid contents of *Solanum lycopersicum*^104^. Moreover, linoleic acid from the dinoflagellate *Karenia mikimotoi* has been shown to block chlorophyll biosynthesis and induce ferroptosis in algal cells^105^. Allelochemicals-ellagic acid, gallic acid, caffeic acid, and p-hydroxybenzoic acid block photosynthetic pigment actions and other critical physiological parameters in *Cylindrospermopsis raciborskii*^106^.

### Allelochemicals impact the essential biochemicals of Mung

**Fig. 3:**
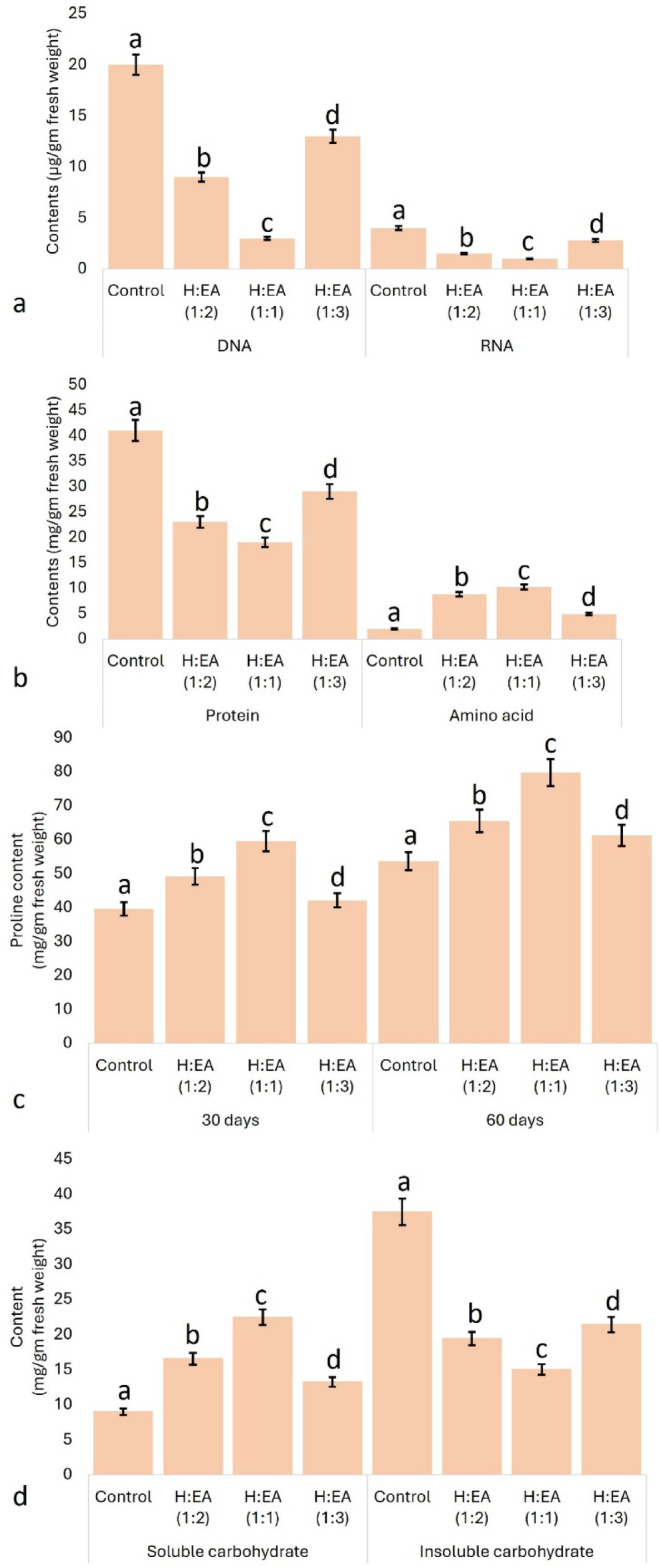
Effect of different fractions of allelochemicals on the **a**- nucleic acid, and **b**- protein-amino acid content of the treated mung bean seeds, and **c**- proline content of treated seedlings, and also **d**- soluble-insoluble carbohydrate content of the seeds. The values in the graph are the mean ± standard error of three independent replicates. The different letters mentioned on the graphs represent the valid statistical differences among them. Tukey’s multiple comparison test was performed to analyse the results (*p* < 0.05).

Extracts of *M*. *suaveolens* reduce the nucleic acid (DNA- 23.19 ± 1.29 and RNA- 3.21 ± 0.09 µg/gm fresh wt.) contents of the treated seeds in comparison to control (DNA- 2.01 ± 0.05 and RNA- 0.62 ± 0.00 µg/gm fresh wt.) seeds (Fig. 3a). The biochemical events are interrupted, and the sugar, as well as protein contents, are reported to be decreased due to the blockage of protein synthesis pathways^107^. The entire cascade of the central dogma is drastically blocked, resulting in a decrease in nucleic acid (i.e., DNA and RNA) material^108^. Cinnamic acid is reported to inhibit the growth of cabbage seedlings by interfering with essential biochemical steps^109^. The allelochemicals interfere with cell division, slowing down or prematurely terminating protein and nucleic acid synthesis^108^. Protein and free amino acid contents are inversely proportional (Fig. 3b). An increase in the concentration of free amino acids is indicative of protein degradation due to treatment with allelochemicals. An improper osmotic adjustment, pH imbalance, and increased ROS population could be the probable causes of the disequilibrium of amino acid-protein contents^110,111^. Allelopathic potentialities of maize toward soybean exhibit a similar pattern to our present results^112^. Different allelopathic plants alter the growth and survival of various receiving plants by disrupting the protein-amino acid equilibrium through the mediation of their emitted or synthesised allelochemicals^113–117^.

Treatment of test plants with allelochemicals leads to a significant accumulation of proline (61.23 ± 2.98 and 97.96 ± 3.46 mg/gm fresh wt.) after thirty and sixty days of treatment compared to control (34.73 ± 1.27 and 48.39 ± 1.48 mg/gm fresh wt.) seedlings (Fig. 3c). Rise in soluble carbohydrates (control- 9.29 ± 0.93 and treated- 21.51 ± 2.37 mg/gm fresh wt.) and a decrease in insoluble carbohydrates were reported during the time of seed germination in comparison to the control set (Fig. 3d). Proline and soluble carbohydrates are potential osmo-regulators that counterbalance chemical stress exerted by allelochemicals^113,118,119^. Allelochemicals of *Melaleuca leucadendra*, *Rumex dentatus*, and *Alhagi maurorum* exhibit similar responses in *Portulaca oleracea*, *Vigna radiata*, and *Pisum sativum*^14,120,121^. Allelochemicals of *Teline monspessulana* and *Ulex europaeus* hamper the carbohydrate metabolism in native Chilean tree species- *Quillaja saponaria* and *Peumus boldus*^122^. Allelochemicals alter the levels of soluble and insoluble carbohydrates and deviate the carbohydrate metabolism of the receiver species^123,124^.

Prolines perform a variety of functions- prevent protein denaturation by balancing the hydration level, activate the Krebs cycle, and initiate chlorophyll biosynthesis. It also constitutes an energy source^125–129^. H: EA (1:1 and 1:2) induces maximum accumulation of these two osmolytes, which indicates that plant cells are trying to balance the negative impact of allelochemicals. The allelopathic effect of foliar extracts of *Heliotropium foertherianum* on radishes satisfies our present reports^130^. Allelochemicals of *Nicotiana plumbaginifolia* perform similar action on *Senna sophera*, *Chenopodium album*, *Senna tora*, and *Setaria viridis*^113^. Allelochemicals like meta-tyrosine, β-cembrenediol, acacetin, coumarin, and β-pinene affect the proline contents of some receiver plants, e.g., *Lactuca sativa*, *Raphanus sativus*, *Allium cepa*, and *Cucumis sativus*^131,132^. Allelochemicals of *Olea europaea* and *Ficus carica* affect the proline contents, activities of phenylalanine and tyrosine ammonia lyase enzymes of *Lactuca sativa*^133^. Allelopathic interaction between *Trianthema portulacastrum* and *Convolvulus arvensis* indicates similar patterns^134^.

The nitrite contents in the control, and treated (1:1, 1:2, and 1:3 H: EA) sets are detected to be 11.237 ± 0.812, 31.759 ± 2.794, 25.412 ± 1.210, and 19.327 ± 1.094 n mol/g fresh weight, respectively. The indole acetic acid and gibberellic acid contents are found to be 98.31 ± 2.67 and 68.35 ± 1.53 ng/g fresh weight in the control plant sets. Whereas, in the treated sets (H: EA-1:1), the values changed to 51.34 ± 2.03 and 32.65 ± 1.35 ng/g fresh weight, respectively. In case of 1:2 and 1:3 H: EA treated sets, the values of indole acetic acid and gibberellin were estimated to be 71.46 ± 2.96, 47.94 ± 1.83, 88.53 ± 3.06 and 53.17 ± 2.17 ng/g fresh weight, respectively.

The enzyme nitrite reductase performs the conversion from nitrite to nitrate in plant cells. Still, the blockage of nitrite reductase (due to the allelochemical application) leads to higher levels of nitrite accumulation in plant tissues^135^. Reports suggest that abiotic stressors, such as water shortages, high temperatures, soil nutrient imbalances, high acidity/basicity, hypoxia, heavy metal contamination, salinity, and root damage, cause elevated nitrite levels^136–141^. The outcomes of^142^ speak about the modulations of nitrite concentration in hypersensitive tobacco plants. The reported allelochemicals primarily interfere with nitrite reduction by modulating the turnover number of nitrite reductase, resulting in a high accumulation of nitrites in the leaves of treated plants^143^. These changes in nitrite concentration indicate disturbances in the plant’s metabolic processes.

Several researchers have demonstrated the elevation of IAA levels in the initial phases of allelochemical treatment, and a gradual decrease in endogenous IAA levels occurs upon prolonged exposure to abiotic stress^144^. Initially, an increase in endogenous IAA levels leads to the modulation of SOD, CAT, and POD levels and thus tries to cope with the ROS, also altering the abscisic acid, cytokinin, jasmonic acid, gibberellic acid, and salicylic acid levels in stress-faceted plant tissues^145^. Reports from^146^ document similar responses while working with *Lepidium draba* L. allelochemicals on *Zea mays*, and *Amaranthus retroflexus*. Similar reactions have been observed by^147^, who found that the application of α-pinene to *Elymus nutans* moderates the levels of ABA, Zeatin, Salicylic acid, gibberellin, Jasmonic acid, and IAA in treated plants. Garlic allelochemical (diallyl disulfide) impacts the growth of cucumber seedlings by interfering with its endogenous plant growth promoter levels^148^.

Various abiotic stress conditions (heavy metal contamination, drought, salinity, soil toxicity) lead to the reduced synthesis of gibberellic acid, and it causes the activation of DELLA protein which leads to the retardation of growth (root-shoot growth, leaf expansion, flowering and fruiting) to conserve energy and ensure plants’ survival or exhibit an escape route in critical phases of environmental stress^117,149,150^. The allelopathic stress induces the elevation of plant growth inhibitor (abscisic acid) in some cases, whereas the jasmonic acid levels increase as a response to elevated stress^151^. The levels of gibberellic acid in leaves of wheat facing abiotic stress were reduced, along with jasmonate, salicylate, trans-zeatin, and its riboside, as well as components such as cis-zeatin and its ribosides, and abscisic acid. This is facilitated by the activation of the GA-degrading gene^152^. So, the change in IAA and gibberellic acid concentration in the treated seedlings indicates that allelochemicals of *M*. *suaveolens* are affecting the plant health. Hayat et al.^153^ reported that garlic allelochemicals modulate the growth of tomato by influencing the endogenous phytohormone concentrations, i.e., IAA, ABA, cytokinin, GBA, by impacting the functions of specific genes, i.e., auxin-responsive protein (IAA2), like-auxin (LAX5), mitogen-activated protein kinase (MAPK7, and MPK2), respiratory burst oxidase homolog (RBOH1), CHI3, SODCC1. Székely and Csengele^154^ reported similar responses when dealing with halophyte-derived allelochemicals and their effects on growth modulation in target plants. Levels of critical phytohormones, i.e., ABA, GA, and IAA, were altered in wheat plants upon treatment with allelochemicals from allelopathic plants, specifically *Chenopodium album*, *C*. *murale*, and *C*. *ambrosioides*, which aligns with our current findings^155^.

### Allelopathic stress hinders the antioxidant defence

**Fig. 4:**
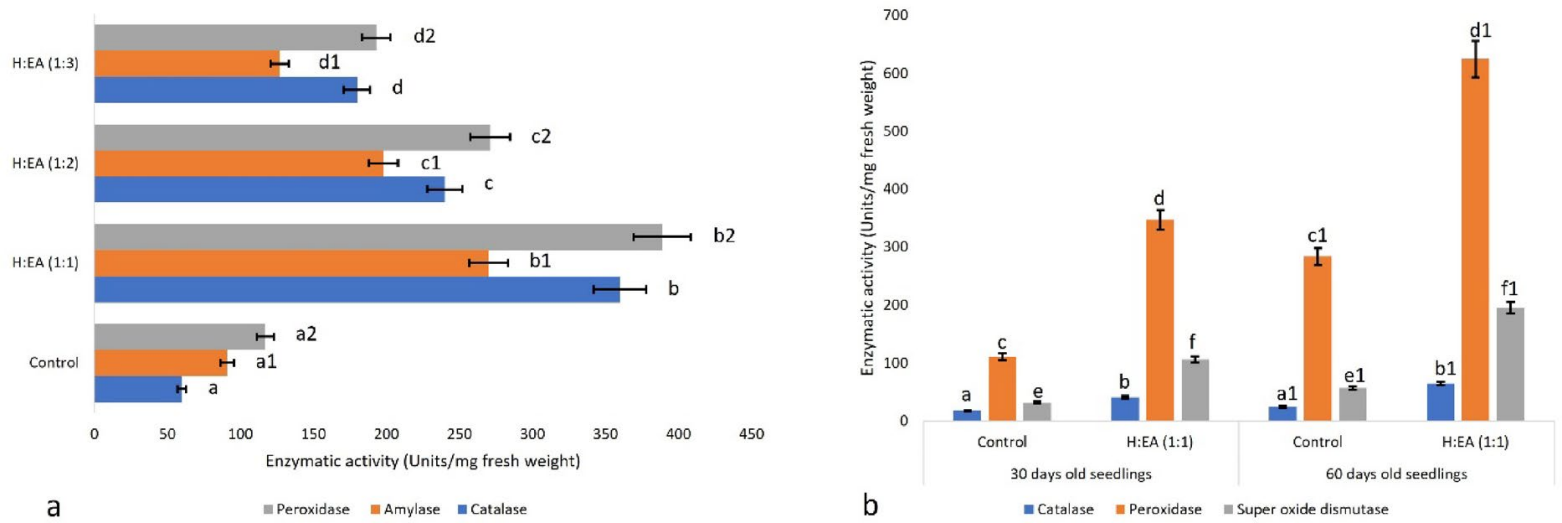
Effect of allelochemicals on enzyme profiles of **a**- mung bean seeds, and **b**- treated mung bean seedlings. The values in the graph are the mean±standard error of three independent replicates. The different letters mentioned on the graphs represent the valid statistical differences among them. Tukey’s multiple comparison test was performed to analyse the results (*p* < 0.05).

An application of allelochemicals to the seeds of mung bean affects their germination, and the necessary biochemical events are blocked. Basic enzymes involved in carbohydrate metabolism, i.e., α-amylase, undergo alterations (Fig. 4a). At the same time, catalase and peroxidase action is enhanced (Fig. 4a), indicating a decrease in PGP (plant growth promoter-gibberellin) activity in the germinating seed. The three major enzymes involved in mitigating oxidative stress within the cellular environment are catalase, peroxidase, and superoxide dismutase^14^. A treatment of mung bean seeds and seedlings with allelochemicals of *M*. *suaveolens* -Hexane: ethyl-acetate (1:1) causes a drastic change of all three necessary enzymes. There were three (treated- 31.67 ± 6.41 and control- 106.33 ± 12.69 units/mg fresh weight) and four (treated- 57.21 ± 8.57 and control- 195.67 ± 15.34 units/mg fresh weight) fold increase in the SOD activity after thirty and sixty days of treatment with allelochemicals in comparison to the control set (Fig. 4b). Peroxidase activity also increased (111.42 ± 11.29 and 346.67 ± 17.31 units/mg fresh weight in the control, 283.67 ± 59.67 and 624.67 ± 63.97 units/mg fresh weight in the 30- and 60-day-treated groups, respectively) drastically in the allelochemical-treated lots. In contrast, the non-treated ones retained their standard enzymatic levels (Fig. 4b). Catalase activity was drastically hampered, and a 2.5-fold increase was found between the control and the allelochemical-treated sets (control: 17.91 ± 1.09 and 24.32 ± 1.43 units/mg fresh weight for 30 and 60 days, respectively; treated: 41.19 ± 2.03 and 64.89 ± 3.17 units/mg fresh weight for 30 and 60 days, respectively) (Fig. 4b). The fourth parameter for evaluating stress responses in the calculation of lipid peroxidation is MDA content analysis. It has been documented that allelochemicals cause a 2-fold increase in lipid peroxidation (control: 17.20 ± 1.9, 21.73 ± 2.11 nmol/g; treated: 30.91 ± 2.71, 52.79 ± 2.96 nmol/g) over the control at 30 and 60 days, respectively.

An increase in MDA content indicates a higher frequency of peroxidation of the plasma membrane due to excessive accumulation of reactive oxygen species (ROS) and altered activity of ROS-scavenging enzymes, such as SOD, POD, and CAT^156^. An increase in SOD activity to detox ROS is simultaneous with the increase in MDA content. The generation of ROS in the cell is a common biochemical event and generally does not cause greater harm, as the cellular antioxidant machinery immediately scavenges them. A treatment with allelochemicals overstresses the plant cell, which disrupts the dynamic equilibrium between ROS generation and removal^157^. The effect of fenugreek extracts on lettuce seedlings supports our present outcome^158^. Membrane damage under the influence of secondary metabolites possesses a high amount of MDA content, which is linked to lipid peroxidation in cucumber and sorghum roots^159^. Allelochemicals increase the accumulation of reactive oxygen species (ROS) in target plants, resulting in detrimental effects^105^. Several reports on the allelopathic effect of different plants towards model plants suggest the same^160^. Allelochemicals of *Asclepias syriaca* exhibit phytotoxic activities against *Amaranthus retroflexus* L., *Chenopodium album* L., and *Iva xanthifolia* Nutt. through and as a result, the primary antioxidant defence enzymes are elevated^161^. Another example is the plant growth-inhibiting activity of *Impatiens glandulifera* Royle against *Sinapis alba* L. and *Raphanus sativus* L., which represents a similar enhancement in antioxidant defence enzymes^162^. Reports from^163^ include those different plants like- *Centella asiatica* (L.) Urb., *Rotala indica* (Willd.) Koehne, *Solanum nigrum* L., *Commelina benghalensis* L., *Marsilea quadrifolia* L., *Ageratum conyzoides* L., *Cynodon dactylon* (L.) pers., *Spilanthes acmella* L., *Heliotropium indicum* L., *Leucas aspera* (Willd.) Link, *Phyllanthus niruri* L., *Sida acuta* Burm.f,, *Mikania micrantha* Kunth, *Polygonum hydropiper* L., *Physalis heterophylla* Nees exhibit allelopathic activity against *Triticum aestivum*, and the major phytotoxicity was demonstrated through the ROS generation, as evidenced by the elevation of the antioxidant defence enzymes. The plant tries to reverse the damage through the action of antioxidant enzymes, but fails in most cases^164^. A potent allelochemical, cinnamic acid at a dosage of 400 mg L^−1^ causes a complete change in SOD action in *Chrysanthemum coronarium*, which matches our present investigational output^165^. A sharp reduction in peroxidase activity hampers the overall health of the seedlings, and a cascade of biochemical events is blocked^166^. POD performs a variety of functions, i.e., regulates the indole acetic acid levels and membrane permeability, induces cell wall formation and disease resistance, modulates oxygen supply to the cells through the seed coat, and maintains seed dormancy^167–171^. Alghanem^114^ documented that leaf leachates of *Aizoon canariense* elevated the antioxidant defence-related enzymes SOD, Catalase, and MDA and hydrogen peroxide contents in four tested seedlings of *Triticum aestivum*, *Hordeum vulgare*, *Brassica napus*, and *Vigna radiata*. Similar outcomes have been reported by Jeddi et al.^172^, where leaf extracts of *Acacia saligna* (Labill.) Wendl. and *Cupressus sempervirens* L. affects the germination and early seedling growth of alfalfa (*Medicago sativa* L.) through modulating the enzymes linked with antioxidant defence. Zhang et al.^166^ also document similar reports on the allelopathic effect of some selected plants on *Medicago sativa*. Phytotoxic effects of *Dactyloctenium aegypticum* on the *Oryza sativa* seedlings operate in the same way by modulating the antioxidant defence systems^160^. Allelochemicals present in the n-butanol extracts of *Cyclachaena xanthiifolia* block the growth and germination of *Brassica juncea* by altering endogenous hormone levels, hampering antioxidant defences, and affecting carotenoid biosynthetic systems, which aligns with our current investigative outputs^117^.

### Characterisation of allelochemicals

**Fig. 5:**
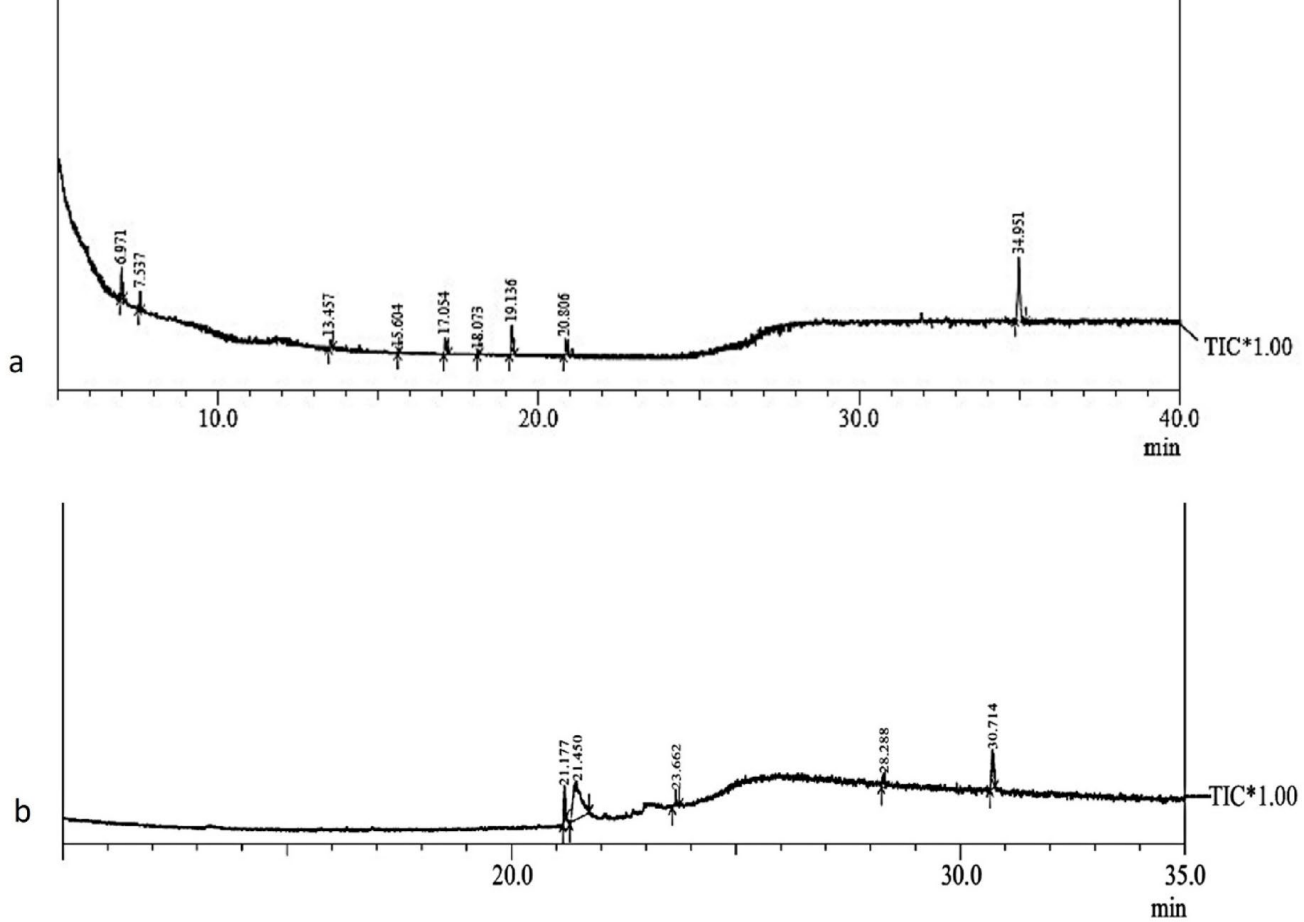
GC-MS chromatogram of the allelochemicals detected in the **a**- H: EA (1:1) and **b**- H: EA (1:2) fractions of methanolic leaf extracts of *M*. *suaveolens*.

Allelochemicals were obtained from the methanolic extract of shade-dried leaves of *M*. *suaveolens*. Methanolic leaf extracts (0.5 g mL^−1^) exhibited maximum seed germination inhibition of 87%. In contrast, the other solvent extracts, i.e., ethanol, chloroform, diethyl ether, n-hexane, and ethyl acetate, exhibited a seed germination inhibition (*V*. *radiata*) of 39%, 43%, 32%, 27%, and 35%, respectively (Supplementary Table 1). Eleven ratios of H: EA were eluted through the column, and the maximum allelopathic activity (78% and 39%) was found upon separation with a 1:1 and 1:2 ratio. The other ratios i.e., 1:9, 2:8, 3:7, 4:6 exhibited a seed germination inhibition of 13%, 15%, 26%, 21%, respectively. Fractions from 1:2 and 1:3 had 39%, and 44% inhibition of germination whereas the ratios 3:1, 7:3, 8:2, and 9:1 exhibited an inhibition of < 10%. The allelochemicals present in the most active fraction (1:1 and 1:2) were identified through GC-MS (Table 2; Fig. 5a, b). 3, 4, 5-trihydroxybenzoic acid has been reported as a potent cyanogenic glycoside from Mediterranean plants^133^. It has also been isolated from *Potamogeton malaianus* and *P*. *maackianus* with strong phytotoxicity against *Microcystis aeruginosa*. Transferulic acid has been reported widely from the shoot and root of different plants, and is strongly allelopathic against annual rye grass-*Lolium rigidum* Gaud^173–175^. 4-hydroxybenzoic acid has previously been reported as an herbicidal agent from rice plants with a cidal activity against mustard green-*Brassica juncea*, and wheat-*Triticum aestivum*^176,177^. Dibutyl phthalate has been detected as an ingredient of allelochemicals in *Glehnia littoralis* Fr., *Ipomoea batatas*, and *Atractylodes lancea*, effective against *Brassica pekinensis* (Lour.), *Bidens pilosa* L., *Galinsoga parviflora* Cav., *Lolium multiflorum* Lam., and *Phalaris minor* Retz^178–180^. Ethyl stearate and various methyl esters have been detected as phytotoxic components^178^. Caryophyllene oxide, and chlorogenic acid are known to be herbicidal in action^147,181–183^. Components like 3-Methylheptyl acetate have no previous record as allelochemicals.

**Table 2:** Major allelochemicals detected in the bioassay-guided fractions F2–F5 from methanolic leaf extracts of *M*. *suaveolens*.

Sl. No	Allelochemicals	Molecular weight (g/mol)	Retention time (min)	Probability (%)
*Fraction- H:EA (1:1)*				
1	3,4,5-trihydroxybenzoic acid (C₇H₆O₅)	170.12	6.97	29.78
2	Transferulic acid (C_10_H_10_O_4_)	194.18	7.53	15.94
3	4-hydroxybenzoic acid (C_7_H_6_O_3_)	138.12	13.45	5.61
4	3-Methylheptyl acetate			
(C_10_H_20_O_2_)	172.26	15.60	6.04	
5	Dibutyl phthalate (C_16_H_22_O_4_)	278.34	17.05	15.87
6	Ethyl stearate (C_20_H_40_O_2_)	312.5	18.07	7.47
7	6,10,14-Trimethyl-2-pentadecanone (C_18_H_36_O)	268.5	19.13	31.01
8	Caryophyllene oxide (C_15_H_24_O)	220.35	20.80	17.86
9	Chlorogenic acid (C_16_H_18_O_9_)	354.31	34.95	89.54
*Fraction-H:EA (1:2)*				
1	Propanoic acid, 3-chloro-,4-formyl phenyl ester (C_10_H_9_ClO_3_)	212.63	21.17	63.24
2	P-coumaric acid (C_9_H_8_O_3_)	164.16	21.45	89.67
3	3,4-dihydroxy benzoic acid (C_7_H_6_O_4_)	154.12	23.66	69.31
4	Mevalonic acid 1,5-lactone (C_12_H_20_O_6_)	260.28	28.28	77.01
5	Sabinene monohydrate (C_10_H_18_O)	154.25	30.71	92.34

Components like p-coumaric acid have previously been reported for their allelopathic activity against a variety of weeds, bloom-forming algae, herbs (*Panax quinquefolius*), *Morchella* mushrooms, and even parasitic plants (*Cuscuta campestris*), etc^123,184–188^. Sabinene has earlier been detected as an allelochemical from various taxa- *Artemisia herba-alba*, *Juniperus horizontalis*,

*Solidago canadensis*, *S. altissima*, *Tanacetum vulgare*, *Satureja calamintha* by several researchers^189–194^. Sabinene monohydrate is a naturally occurring bicyclic monoterpene and a significant part of most of the essential oils obtained from plants. *M*. *suaveolens* is a member of Lamiaceae, and different members, i.e., *Calamintha* sp., *Coleus* sp., *Conradina* sp., *Satureja* sp., *Tectona* sp., *Orthosiphon* sp., *Hyssopus* sp., *Rosamarinus* sp., *Origanum* sp., *Leonurus* sp., *Leucas* sp., *Hyptis* sp., *Thymus* sp., *Ocimum* sp., *Lavendula* sp., *Nepeta* sp., *Salvia* sp., of this family are potent allelochemical-producing plants^192^. Allelochemicals from invasive alien species comprise diverse groups of chemicals (phenolics, terpenoids, alkaloids, and fatty acids, etc.) and interfere with various physiological functions^195–198^. Invasive taxon *Senna spectabilis*, *Acacia melanoxylon*, *Eucalyptus globulus*, *Ulex europaeus*, *Ageratina Adenophora*, *Pueraria montana* var. *lobata* blocks the growth of native species- *Ailanthus tryphysa*,* Pongamia pinnata*,* Tectona grandis*,* Hopea parviflora*,* Dendrocalamus strictus*, *Acacia dealbata*,* Phytolacca americana*, *Lactuca sativa*, *Pinus roxburghii* and synthesises metabolites- Neophytadiene, Nerolidol, and 2,4-Ditert-Butylphenol, trihydroxyoctadecenoic acid, tyrosol glucoside, gentistic acid, scopoletin, quinolizidine alkaloids, xanthoxins, *p*-coumaric acid, caffeic acid, methyl caffeate and daidzein^198–201^.

### Relative phytotoxicity of the currently investigated allelochemicals with standard compounds and synthetic herbicides

Out of the fourteen allelochemicals reported through spectroscopic techniques, the standard compounds of the major ones, i.e., sabinene and p-coumaric acid (from the 1:2 fraction) and 3,4,5-trihydroxybenzoic acid, transferulic acid, dibutyl phthalate, and chlorogenic acid (from the H: EA-1:1 fraction), are bought from the market. A mixture was prepared following their natural occurrence as detected from GC-MS data. The four metabolites from the 1:1 fraction were mixed in a 3,4,5-trihydroxybenzoic acid: Transferulic acid: Dibutyl phthalate: Chlorogenic acid − 1:1:2:5 ratio, which caused an inhibition of 56%. P-coumaric acid and sabinene (as detected in the H: EA-1:2 fraction) were mixed in a 1:1 ratio with an inhibition of 23%. A combination of all six of these metabolites caused an inhibition of 79%. Thus, these six major allelochemicals reported from the present study can be employed as effective herbicidal agents, supporting the motto of sustainable agricultural practices.

The synthetic herbicide, Excel Mera 71, a formulation with ammonium salt of Glyphosate, was used (0.1 mg mL^− 1^), and an inhibition of 92% was recorded. In contrast, the *M*. *suaveolens* allelochemicals (0.5 mg mL^−1^) exhibit 79% inhibition of Mung bean seed germination. Upon co-application of 0.5 mg mL^− 1^ of allelochemicals (both the fractions) with 0.05 mg mL^− 1^ of Excel Mera 71, there was a mung bean seed inhibition of 98% which highlights the synergistic effect of the allelochemicals with the synthetic herbicide. Upon proper channelization, these *M*. *suaveolens* allelochemicals may reduce the requirement of per-acre synthetic herbicides to tackle weeds.

### Molecular docking analysis between allelochemicals and auxin-binding protein 1 (PDB-ID 1LRH), 4-hydroxyphenylpyruvate dioxygenase (6J63), and tryptophan synthase beta-subunit (5dw3)

Docking of allelochemicals with the target receptor 1LRH (Table 3) exhibits binding affinity of − 6.325 kcal/mol (Supplementary Fig. 1). The other receptors 5DW3 and 6J63 exhibit a binding energy of − 6.24, − 6.915 (Tables 4 and 5). Specific interactions between allelochemicals and amino acid residues within the active site of the 1LRH, 5DW3, and 6J63 suggest their involvement in receptor-ligand binding (Supplementary Figs. 2 and 3). Auxin Binding Protein 1 (ABP1), a common herbicide target, is involved in critical cellular responses to auxin, i.e., cellular elongation, cell division, plasma membrane hyperpolarisation, and protoplasmic ion flux^]^. 5DW3 represents tryptophan synthase β-subunit, which catalyses the ultimate phase in the biosynthesis of L-tryptophan. It remains as an alpha-beta-beta-alpha complex of heteroenzyme. The alpha subunit produces indole, which diffuses through a tunnel to the beta subunit’s active site to produce tryptophan with L-serine. 4-hydroxyphenylpyruvate dioxygenase is involved in tyrosine catabolism and the synthesis of plastoquinone and tocopherol, which function to oxidise 4-hydroxyphenylpyruvic acid to homogentisic acid, which acts as a precursor. It is also a prime target for herbicide design, like the other two enzymes mentioned earlier. Its inhibition leads to bleaching in plants.

**Table 3:** Representation of the binding energy of different allelochemicals with 1LRH.

Allelochemicals	Pub Chem ID	Binding energy (kcal/mole)	Interactions involved
P-coumaric acid	637,542	− 5.449	Hydrophobic Interaction [Leu161(A)-3.52] Hydrogen Bond [Thr105(A)-2.73, Ala107(A)-2.08, Gly108(A)-2.87, Gly228(A)-2.17, Gly228(A)-2.36, Pro297(A)-2.20, Gly298(A)-3.23]
3,4-dihydroxybenzoic acid	72	− 6.009	Hydrophobic Interaction [Thr160(A)-3.72, Lys162(A)-3.72, Pro291(A)-3.89] Hydrogen Bond [Ile289(A)-2.44] Salt Bridge [Lys162(A)-4.81]
Propanoic acid, 3-chloro-, 4-formyl phenyl ester	578,360	− 4.813	Hydrophobic Interaction [Ala81(A)-3.74, His204(A)-3.14, Ala113(A)-3.83, Gly229(A)-1.97] Hydrogen Bond [Ala81(A)-1.74, Gln109(A)-3.14, Arg204(A)-3.43, Gly228(A)-2.21, Glu229(A)-1.95, Ser230(A)-2.76, Arg271(A)-1.43]
Mevalonic acid 1, 5-lactone	45,109,791	− 4.394	Hydrophobic Interaction [His110(A)-3.81, Ala113(A)-3.83, Ala343(A)-3.11, Glu345(A)-3.91] Hydrogen Bond [Ala86(A)-1.97, Gln109(A)-3.40, Gln109(A)-3.50, Gly227(A)-1.71, Gly228(A)-2.21, Gly229(A)-1.81] π-Stacking (perpendicular) [His82(A)-4.76] Salt Bridge [His80(A)-2.91]
Sabinene	10,887,971	− 5.702	Hydrophobic Interaction Phe381(A)-3.74, Leu39(A)-3.14, Arg204(A)-3.43 Hydrogen Bond Glu394(A)-2.95 π-Stacking (parallel) Phe379(A)-3.71
3,4,5-trihydroxybenzoic acid	370	− 5.311	Hydrophobic Interaction [Leu39(A)-3.98, Phe199(A)-3.33] Hydrogen Bond [Asn36(A)-3.05, Ser201(A)-3.39, Val202(A)-2.14] Salt Bridge [Arg205(A)-4.86]
Transferulic acid	445,858	− 5.427	Hydrophobic Interaction [Pro297(A)-3.74, Ile380(A)-3.81, Ile380(A)-3.43] Hydrogen Bond [Arg136(A)-2.95, Arg136(A)-2.54, Arg136(A)-3.09] Salt Bridge [Lys383(A)-5.46]
4-hydroxybenzoic acid	135	− 4.586	Hydrophobic Interaction [Leu39(A)-3.64, Phe199(A)-3.58] Hydrogen Bond [Asn36(A)-2.24, Arg205(A-3.52)] π-Cation Interaction [Arg205(A)-5.05]
3-Methylheptyl acetate	537,686	− 4.267	Hydrophobic Interaction [Leu39(A)-3.72, Leu43(A)-3.72, Asp198(A)-3.86, Val202(A)-3.96] Hydrogen Bond [Asp198(A)-2.87, Ser201(A)-2.85] Salt Bridge [Arg205(A)-4.81]
Dibutyl phthalate	3026	− 5.292	Hydrophobic Interaction [Lys30(A)-3.77, Phe35(A)-3.99, Leu39(A)-3.51, Asp198(A)-3.70, Phe199(A)-3.66, Phe199(A)-3.48, Val202(A)-3.75] Salt Bridge [Arg205(A)-4.27, Arg205(A)-4.39]
Ethyl stearate	8122	− 5.09	Hydrophobic Interaction [Val11(A)-3.57, Leu161(A)-3.57, Tyr301(A)-3.76, Gly269(A)-3.57]
6,10,14-Trimethyl-2-pentadecanone	10,408	− 6.098	Hydrophobic Interaction [Leu161(A)-3.57, Leu161(A)-3.50, Ile165(A)-3.70, Gly298(A)-3.66, Tyr301(A)-3.76] Hydrogen Bond [His110(A)-2.19]
Caryophyllene oxide	1,742,210	− 5.19	Hydrophobic Interaction [Val11(A)-3.92, Pro12(A)-3.65, Leu15(A)-3.66, Leu169(A)-3.78] Hydrogen Bond [Arg170(A)-2.66, Arg170(A)-2.57]
Chlorogenic acid	1,794,427	− 6.325	Hydrophobic Interaction [Leu43(A)-3.06, Val202(A)-3.37, Val202(A)-3.66] Hydrogen Bond [Arg49(A)-3.05, Asp198(A)-2.70] π-Cation Interaction [Arg205(A)-4.77]

**Table 4:** Representation of the binding energy of different allelochemicals with 6J63 receptor (4-hydroxyphenylpyruvate dioxygenase).

Allelochemicals	Binding energy (kcal/mole)	Interactions involved
P-coumaric acid	− 5.449	Hydrophobic Interaction Phe381(A)-3.65, Phe392(A)-3.85, Phe424(A)-3.79 Hydrogen Bond Glu394(A)-2.44 π-Stacking (parallel) Phe381(A)-3.88 Salt Bridge His226(A)-4.56, His308(A)-4.15
3,4-dihydroxybenzoic acid	− 5.104	Hydrophobic Interaction Phe381(A)-3.37 Hydrogen Bond Asn282(A)-1.73, Asn282(A)- 2.60, Gln307(A)-3.11, Gln379(A)- 2.31, Phe419(A)-2.43, Gly420(A)- 2.87, Lys421(A)-2.47 π-Stacking (perpendicular) Phe424(A)-5.22 Salt Bridge His308(A)-4.60
Propanoic acid, 3-chloro-, 4-formyl phenyl ester	− 4.813	Hydrophobic Interaction Phe381(A)-3.31 Phe419(A)-1.79 Hydrogen Bond Glu282(A)-1.87
Mevalonic acid 1, 5-lactone	− 4.394	Hydrophobic Interaction Met331(A)-3.67, Thr102(A)-3.82, Tyr268(A)-2.03 Hydrogen Bond Ala88(A)-2.03
Sabinene	− 5.702	Hydrophobic Interaction Phe381(A)-3.03, Phe392(A)-2.50 Hydrogen Bond Pro103(A)-2.49 π-Stacking (parallel) Phe108(A)-4.88
3,4,5-trihydroxybenzoic acid	− 5.079	Hydrophobic Interaction [Ala101(A)-3.82] Hydrogen Bond [Ala57(A)-2.76, Thr58(A)-2.65, Tyr88(A)-2.03, Tyr88(A)-2.35, Pro102(A)-2.59, Tyr103(A)-3.34] π-Stacking (perpendicular) [Tyr103(A)-5.18]
Transferulic acid	− 4.983	Hydrophobic Interaction [Met335(A)-3.77, Leu368(A)-3.76, Leu368(A)-3.64, Phe381(A)-3.42, Leu427(A)-3.75]
4-hydroxybenzoic acid	− 5.289	Hydrophobic Interaction [Phe419(A)-3.55] Hydrogen Bond [Ser267(A)-1.90, Asn282(A)-2.65, Glu394(A)-1.95]
3-Methylheptyl acetate	− 3.852	Hydrophobic Interaction Phe335(A)-3.42, Phe392(A)-3.75, Phe424(A)-3.79 Hydrogen Bond Glu394(A)-2.65
Dibutyl phthalate	− 4.084	Hydrophobic Interaction [Val228(A)-3.69, Leu265(A)-3.78, Pro280(A)-3.90, Pro280(A)-3.97, Phe381(A)-3.79, Phe419(A)-3.61, Phe424(A)-3.78, Phe424(A)-3.74, Phe424(A)-3.71] Salt Bridge [His308(A)-5.04, His308(A)-5.00]
Ethyl stearate	− 4.051	Hydrophobic Interaction [Val226(A)-3.69, Leu261(A)-3.78, Phe381(A)-3.79, Phe419(A)-3.61, Phe424(A)-3.78, Phe424(A)-3.61, Phe424(A)-3.74]
6,10,14-Trimethyl-2-pentadecanone	− 4.916	Hydrophobic Interaction [Ile162(A)-3.87, Lys180(A)-3.90, Tyr182(A)-3.55, Val231(A)-3.89, Tyr240(A)-3.77, Tyr240(A)-3.57] Hydrogen Bond [Gly183(A)-3.50]
Caryophyllene oxide	− 6.915	Hydrophobic Interaction Val269(A)-2.69, Phe180(A)-3.79, Tyr182(A)-2.57 Hydrogen Bond Asn128(A)-1.11, Asn182(A)- 2.03, Gly107(A)-1.11, Gln379(A)- 2.31, Phe419(A)-2.03, Gly420(A)- 2.87, Lys121(A)-2.11 π-Stacking (perpendicular) Phe419(A)-5.60 Salt Bridge His108(A)-4.22
Chlorogenic acid	− 5.312	Hydrophobic Interaction Phe182(A)-2.11, Tyr180(A)-3.82 Hydrogen Bond Asn128(A)-1.11, Asn182(A)- 2.03, Gly107(A)-1.11, Gln379(A)- 2.31, Phe419(A)-2.03, Gly420(A)- 2.87, Lys121(A)-2.11

**Table 5:** Representation of the binding energy of different allelochemicals with Tryptophan Synthase beta-subunit (5DW3).

Allelochemicals	Binding energy (kcal/mole)	Interactions involved
P-coumaric acid	− 5.449	Hydrophobic Interaction [Ala79(A)-2.75, Gln110(A)-2.19, Ala114(A)-2.73, Ala343(A)-3.73, Gln341(A)-1.87] Hydrogen Bond [Gln46(A)-2.07, Gly109(A)-4.34, Ala119(A)-2.73, 227(A)-1.71, His228(A)-1.21, Ala371(A)-1.97] π-Stacking (perpendicular) [His81(A)-4.66] Salt Bridge [His81(A)-4.91]
3,4-dihydroxybenzoic acid	− 4.794	Hydrophobic Interaction Val80(A)-3.75, His110(A)-3.81, Ala113(A)-3.83, Ala283(A)-2.07, Glu345(A)-2.02 Hydrogen Bonds [Ala86(A)-1.97, Gln109(A)-3.40, Gln109(A)-3.50, Gly227(A)-1.71, Asn231(A)-2.14] π-Stacking (parallel) His89(A)-2.81
Propanoic acid, 3-chloro-, 4-formyl phenyl ester	− 4.813	Hydrophobic Interaction [Ala80(A)-2.14, His113(A)-3.81, Ala113(A)-3.83, Ala343(A)-3.73, Glu345(A)-3.97] Hydrogen Bond [Gln27(A)-2.09, Gly228(A)-2.21, Gly229(A)-1.81, Ser230(A)-2.76, Asn231(A)-2.14, Ser371(A)-1.80]
Mevalonic acid 1, 5-lactone	− 4.394	Hydrophobic Interaction [Ala78(A)-2.37, His109(A)-4.19, Ala110(A)-3.71, Ala139(A)-2.73, Glu345(A)-3.97] Hydrogen Bond [Ala79(A)-2.07, Gln111(A)-3.43, Glu101(A)-2.50, Gly227(A)-1.71] Salt Bridge [Ala81(A)-4.97]
Sabinene	− 5.702	Hydrophobic Interaction [Ala77(A)-3.17, His103(A)-2.48, Ala136(A)-4.14, Glu137(A)-2.07, Gly119(A)-3.92]
3,4,5-trihydroxybenzoic acid	− 5.164	Hydrophobic Interaction [His109(A)-2.55, Ala109(A)-3.73, Ala319(A)-3.79, Glu387(A)-4.07] Hydrogen Bond [Ala87(A)-1.07, Glu118(A)-1.73, Gln119(A)-2.48, Gly245(A)-1.69, Asn261(A)-2.93]
Transferulic acid	− 5.236	Hydrophobic Interaction [His117(A)-4.07, Ala119(A)-1.93, Ala343(A)-1.62, Glu447(A)-4.27] Hydrogen Bond [Ala67(A)-1.58, Gly231(A)-1.23, Ser230(A)-2.76, Asn231(A)-3.07, Ser319(A)-1.93] π-Stacking (perpendicular) [His79(A)-2.21] Salt Bridge [His81(A)-3.17]
4-hydroxybenzoic acid	− 5.342	Hydrophobic Interaction [Ala71(A)-2.34, His117(A)-2.91, Ala111(A)-2.09, Ala343(A)-1.03] Hydrogen Bond [Ala79(A)-2.09, Glu229(A)-1.78, Gly231(A)-1.23, Ser207(A)-3.16, Asn219(A)-3.91] π-Stacking (parallel) [Ser201(A)-3.76]
3-Methylheptyl acetate	− 4.538	Hydrophobic Interaction [Ala80(A)-3.15, His110(A)-3.92, Ala137(A)-4.17, Ala349(A)-2.14, Glu325(A)-4.21] Hydrogen Bond [Ala86(A)-1.97, Gln109(A)-3.40, Gln109(A)-3.50, Gly227(A)-1.71, Ser371(A)-1.80]
Dibutyl phthalate	− 4.395	Hydrophobic Interaction [Ala80(A)-3.75, Ala343(A)-3.73, Glu345(A)-3.97] Hydrogen Bond [Gly229(A)-1.81, Ser230(A)-2.76]
Ethyl stearate	− 3.69	Hydrophobic Interaction [Ala79(A)-2.97, Gly119(A)-1.83, His230(A)-3.50]
6,10,14-Trimethyl-2-pentadecanone	− 3.851	Hydrophobic Interaction [His107(A)-2.74, Ala103(A)-1.74, Ala434(A)-2.93, Glu345(A)-3.97] Hydrogen Bond [Ala86(A)-1.97, Gln109(A)-3.40, Gln109(A)-3.50, Gly227(A)-1.71] π-Stacking (perpendicular) [Gln101(A)-2.46]
Caryophyllene oxide	− 5.673	Hydrophobic Interaction [Ala70(A)-3.05, His116(A)-3.71, Ala115(A)-4.23, Ala123(A)-3.91, Glu315(A)-1.97] Hydrogen Bond [Ala16(A)-2.04, Gly221(A)-1.32, Ser207(A)-1.72, Asn131(A)-2.09, Ser171(A)-1.35] Salt Bridge [Gly(A)-1.29]
Chlorogenic acid	− 6.24	Hydrophobic Interaction [Ala80(A)-3.75, His110(A)-3.81, Ala113(A)-3.83, Ala343(A)-3.73, Glu345(A)-3.97] Hydrogen Bond [Ala76(A)-2.34, Glu89(A)-2.94, Gly125(A)-2.75, Gly192(A)-1.38] π-Stacking (parallel) [His81(A)-5.70]

ABP1 (1LRH) is a commonly targeted receptor protein by the herbicides and is ubiquitously present in green plants^203^. A recent report from *Forsskaolea tenacissima* allelochemicals (majorly essential oils- β-eudesmol, bulnesol, isolongifolol, chavicol, hexahydrofarnesyl acetone) highlights the ABP1 and HPPD-4-hydroxyphenylpyruvate dioxygenase (PDB ID-6J63) inhibiting activity in target plants- *Dactyloctenium aegypticum*, *Bidens pilosa*^204^. The maximum binding affinities of − 6.95 and − 6.48 were determined from geranylgeraniol against HPPD and ABP1, respectively. Other common targets for herbicides include *Arabidopsis* Dehydroascorbate Reductase 1 (PDB-ID-DHAR1), where chlorogenic acid, a common allelochemical, can bind and block the seedling growth^205^. Allelochemicals (3,4-dihydroxy benzoic acid, vanillic acid, ferulic and succinic acid, etc.) from *Vicia faba* are known to block the tryptophan synthase β subunit of *Pyrococcus furiosus* and are phytotoxic against *Lens culinaris*^206^. They reported the maximum binding affinity of luteolin and naringenin with a value of − 9.2 kcal/mole. The present outcome holds promise for the development of an eco-friendly weed-controlling agent that, singly or in combination with contemporary herbicides, may open sustainable opportunities. A detailed field trial is further required, along with an assessment of the particular toxicological and clinical evaluation for proper utilization of the allelochemical as a bio-weedicide.

## Conclusion

This study shows that *M*. *suaveolens* extract has strong natural weed-suppressing (allelopathic and bioherbicidal) effects on mung bean (*Vigna radiata*) seedlings and the seeds of *Parthenium hysterophorus* and *Sorghum bicolor*. When these plants were exposed to allelochemicals, their growth and vigour dropped rapidly with clear changes in their antioxidant activity, osmolyte levels, pigments and other key metabolic processes. These combined effects make it clear *M*. *suaveolens* has significant phytotoxic potential. From the hexane-ethyl acetate fractions (1:1 and 1:2), fourteen major allelochemicals were identified. Among them, 3,4,5-trihydroxybenzoic acid, trans-ferulic, 4-hydroxybenzoic acid, 3-methylheptyl acetate, p-coumaric acid, mevalonic acid 1,5-lactone, and sabinene monohydrate found as the most influential compounds. The research studies suggest that *M*. *suaveolens* could serve as a safer, eco-friendly alternative to conventional chemicals herbicides. Plant based natural product can help lower the chemical burden in agriculture, reduce environmental pollution, and support healthier food production with less exposure to toxic agrochemicals. In the future, bioherbicide made from *M*. *suaveolens* could be incorporated into sustainable farming systems, that can help to maintain soil health, protect ecosystems, and promote a clearer agricultural environment. More extensive field studies and safety evaluations are needed before it can be recommended for large scale use, to ensure that poses no risk to the environment or human health.

### Future outcomes

Further field-based trials, through intercropping and co-application with minimal dosages of synthetic herbicides, are required to gradually replace or reduce the dosage of synthetic allelochemicals and go for large-scale application. The target-specific approaches, seasonal responses, and economic feasibility of these allelochemicals as novel bioherbicides, as a tool for sustainable development, are the probable future outcomes of the current investigation.

### Limitation

All experiments were performed under laboratory and greenhouse conditions, but on-field validation, where multiple interacting factors are involved, has not been assessed. GC-MS-based identification needs further LC-MS or NMR-based studies. Also, the effect of these allelochemicals needs to be checked in different targeted and non-targeted hosts other than these three studied. Regarding in silico validation, more receptors need to be evaluated for their interaction with the allelochemical. The transcriptomic and proteomic analyses of the treated plants in reference to the control set need to be evaluated. The impact of these allelochemicals on the soil microbial population also needs to be assessed.

## Supplementary Information

Below is the link to the electronic supplementary material.

Supplementary Material 1

## Data Availability

The raw data and biological materials are available. The datasets used and/or analysed during the current study are available from the corresponding author on reasonable request.

## Permission for collection of plant materials-

The host institute and its researchers are eligible to collect the plant materials, *M*. *suaveolens*, from the surroundings as per the Institutional Ethical Guidelines.

## Statement on voucher specimen availability-

The voucher specimen in the form of a herbarium of the plant material, i.e., *M*. *suaveolens*, is deposited in the Departmental herbarium collection of Midnapore College Autonomous with an accession number of MCA-BOT-113, respectively. The information on herbarium sheets will be shared upon request. These taxa were identified by the renowned taxonomist Prof. Gour Gopal Maity, a retired professor at Kalyani University, West Bengal, India.
