# Supplementary material for: Evaluation of allelopathic potentialities of an invasive taxon, Mesosphaerum suaveolens (L.) Kuntze, and bio-assay-guided identification of the involved allelochemicals
Source: Sci Rep. 2026 Mar 26;16:15152. doi: 10.1038/s41598-026-43350-w (PMC13171910; doi:10.1038/s41598-026-43350-w)
Supplement: Supplementary file 1 — Supplementary Material 1 [file 41598_2026_43350_MOESM1_ESM.docx]

**
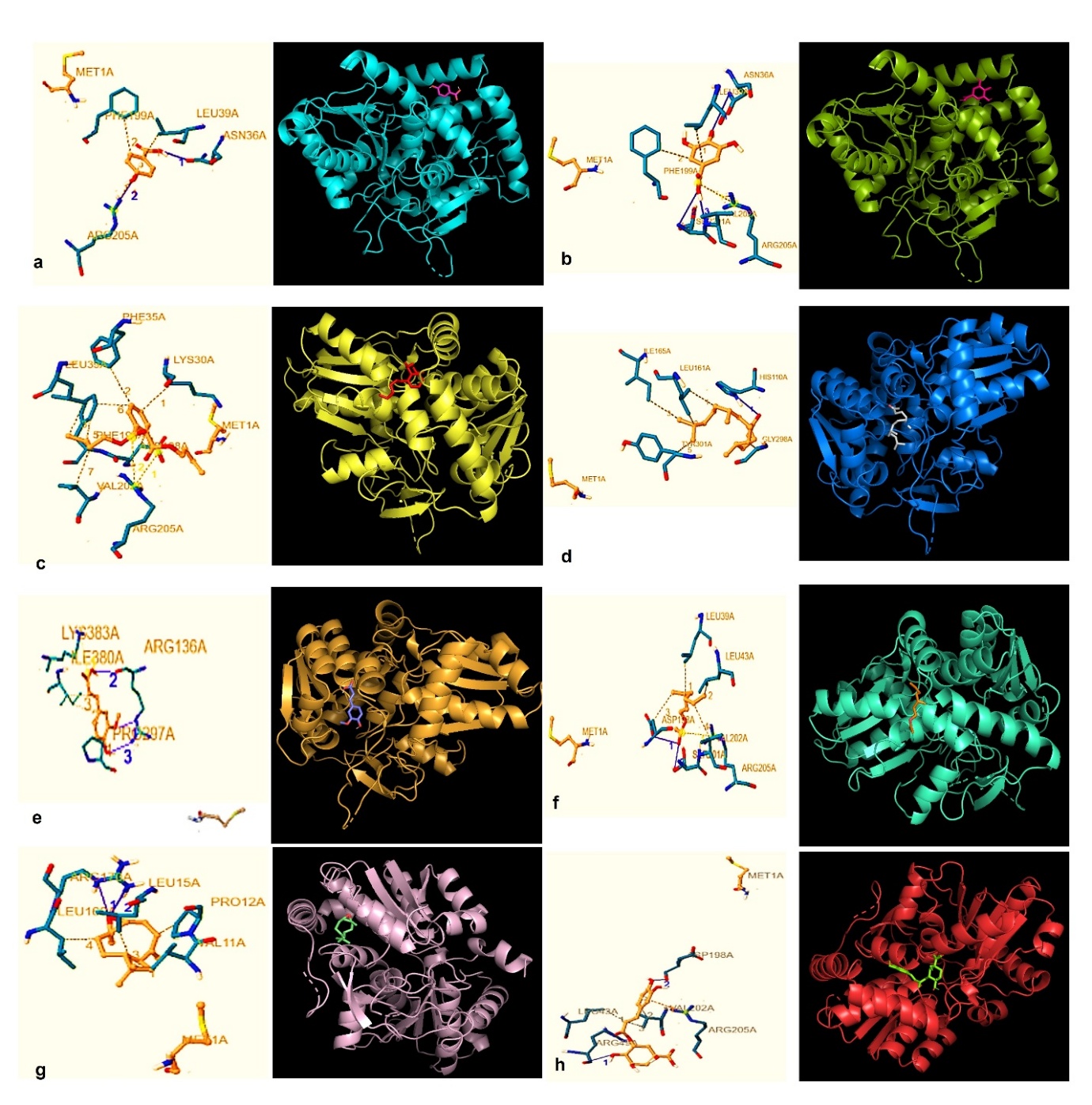
**

**Supplementary Figure 1-** 3D visualization of allelochemicals (ligands) a- 4-hydroxybenzoic acid (Pub Chem ID-135), b- 3,4,5-trihydroxybenzoic acid (370), c- Dibutyl phthalate (3026), d- 6,10,14-Trimethyl-2-pentadecanone (10408), e- Transferulic acid (445858), f- 3-Methylheptyl acetate (537686), g- Caryophyllene oxide (1742210), h- Chlorogenic acid (1794427) binding with the receptor of Auxin Binding Protein 1 (ABP1)-(PDB ID-1LRH).

**
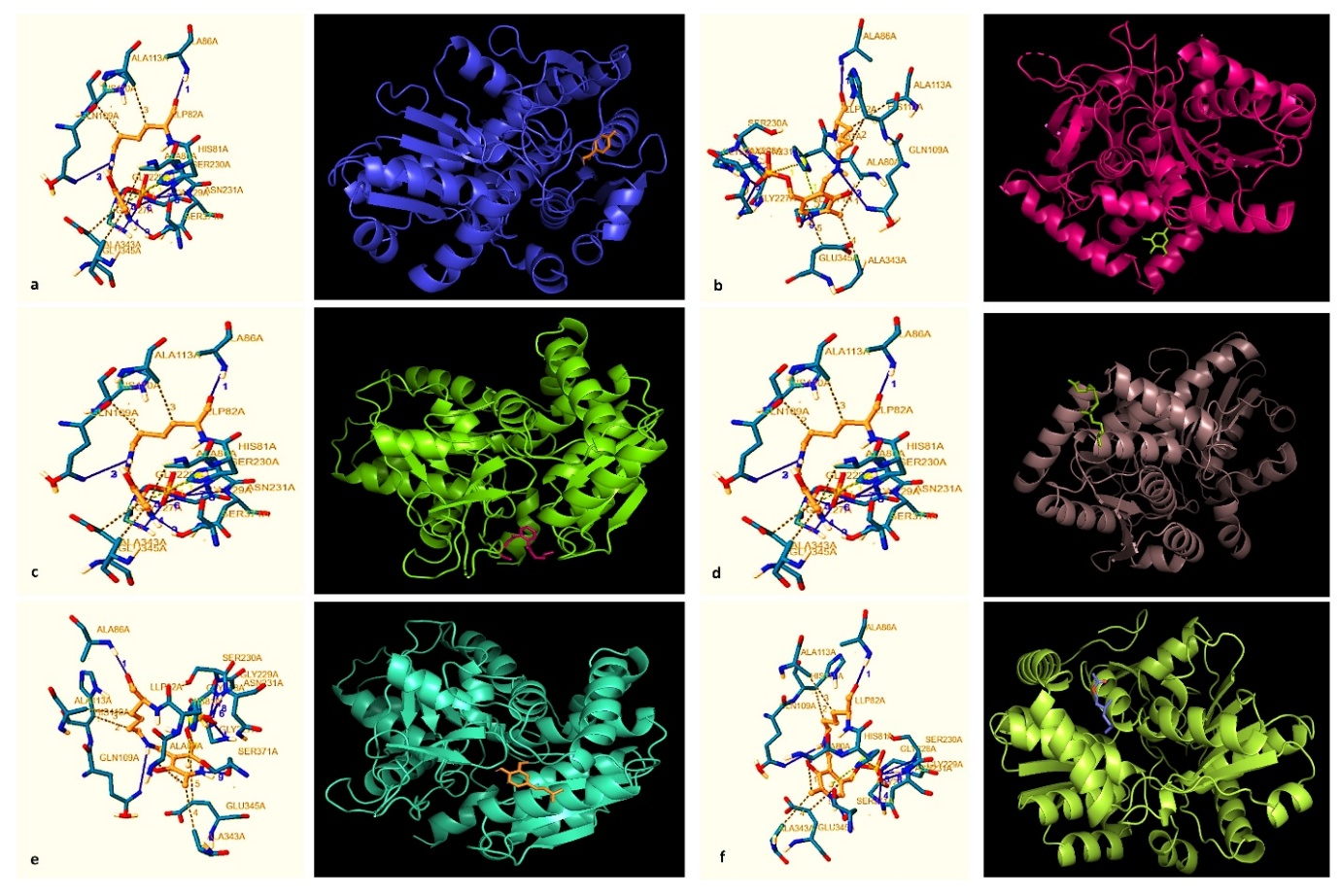
**

**Supplementary Figure 2-** 3D visualization of allelochemicals (ligands) a- 4-hydroxybenzoic acid (Pub Chem ID-135), b- 3,4,5-trihydroxybenzoic acid (370), c- Dibutyl phthalate (3026), d- 6,10,14-Trimethyl-2-pentadecanone (10408), e- Transferulic acid (445858), f- 3-Methylheptyl acetate (537686) binding with the receptor of Tryptophan synthase β-subunit (PDB ID-5DW3).


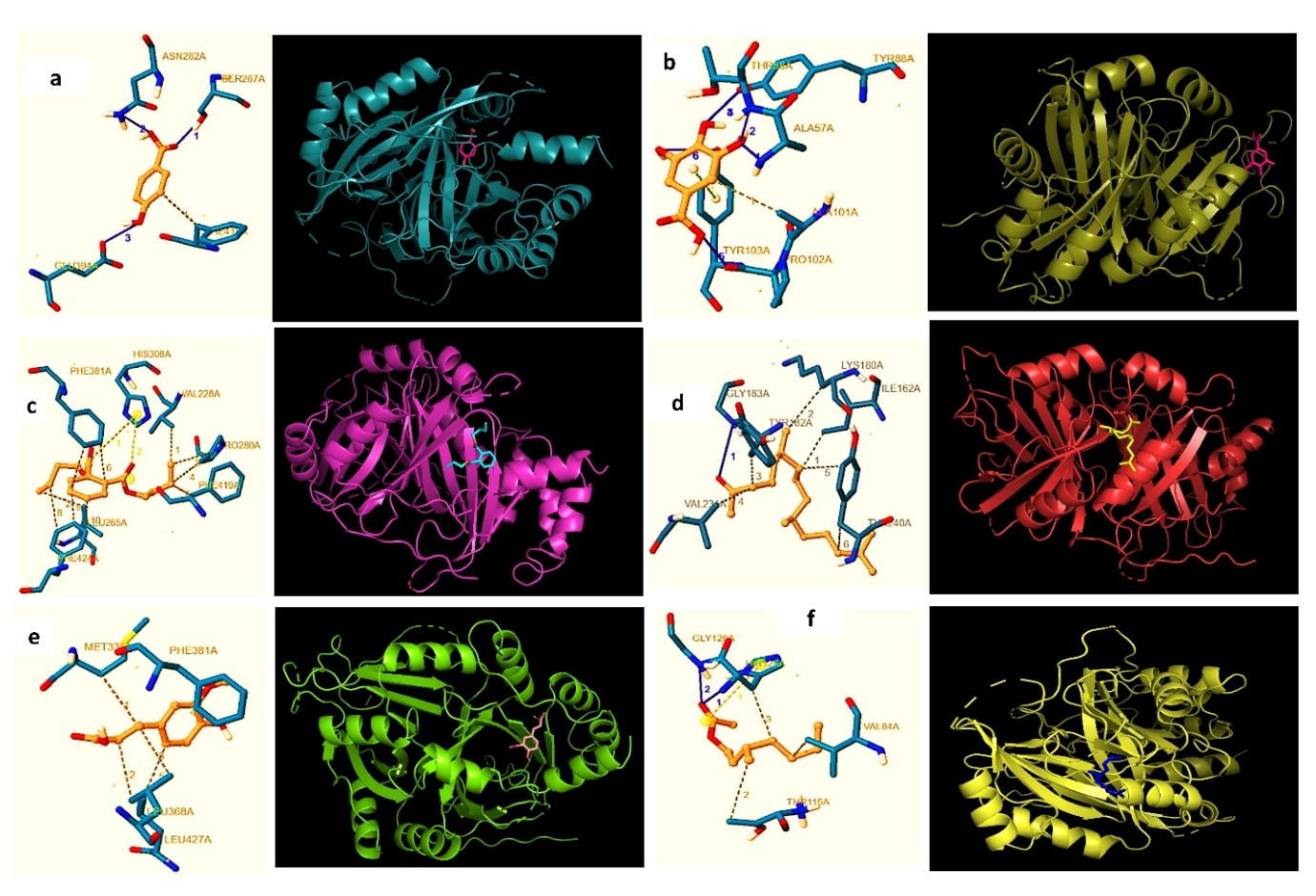


**Supplementary Figure 3-** 3D visualization of allelochemicals (ligands) a- 4-hydroxybenzoic acid (Pub Chem ID-135), b- 3,4,5-trihydroxybenzoic acid (370), c- Dibutyl phthalate (3026), d- 6,10,14-Trimethyl-2-pentadecanone (10408), e- Transferulic acid (445858), f- 3-Methylheptyl acetate (537686) binding with the receptor of 4-hydroxyphenyl pyruvate dioxygenase (PDB ID-6J63).

Supplementary Table 1- Seed (*Vigna radiata*) germination inhibition exerted by different solvent extracts of *M*. *suaveolens*

| **Sl. No.** | **Different solvent extracts** | **Inhibition percentage** |
| --- | --- | --- |
| 1 | Ethanol | 39 |
| 2 | Chloroform | 43 |
| 3 | Diethyl ether | 32 |
| 4 | n-hexane | 27 |
| 5 | Ethyl acetate | 35 |
